# Supplementary material for: Encoding of long-term associations through neural unitization in the human medial temporal lobe
Source: Nat Commun. 2018 Oct 22;9:4372. doi: 10.1038/s41467-018-06870-2 (PMC6197188; doi:10.1038/s41467-018-06870-2)
Supplement: Supplementary file 1 — Supplementary Information [file 41467_2018_6870_MOESM1_ESM.pdf]

Supplementary Information for “Encoding of long-term associations through neural unitization in the human medial temporal lobe”

Rey et al.

This PDF includes:

Supplementary Figures 1 to 9

Supplementary Table 1

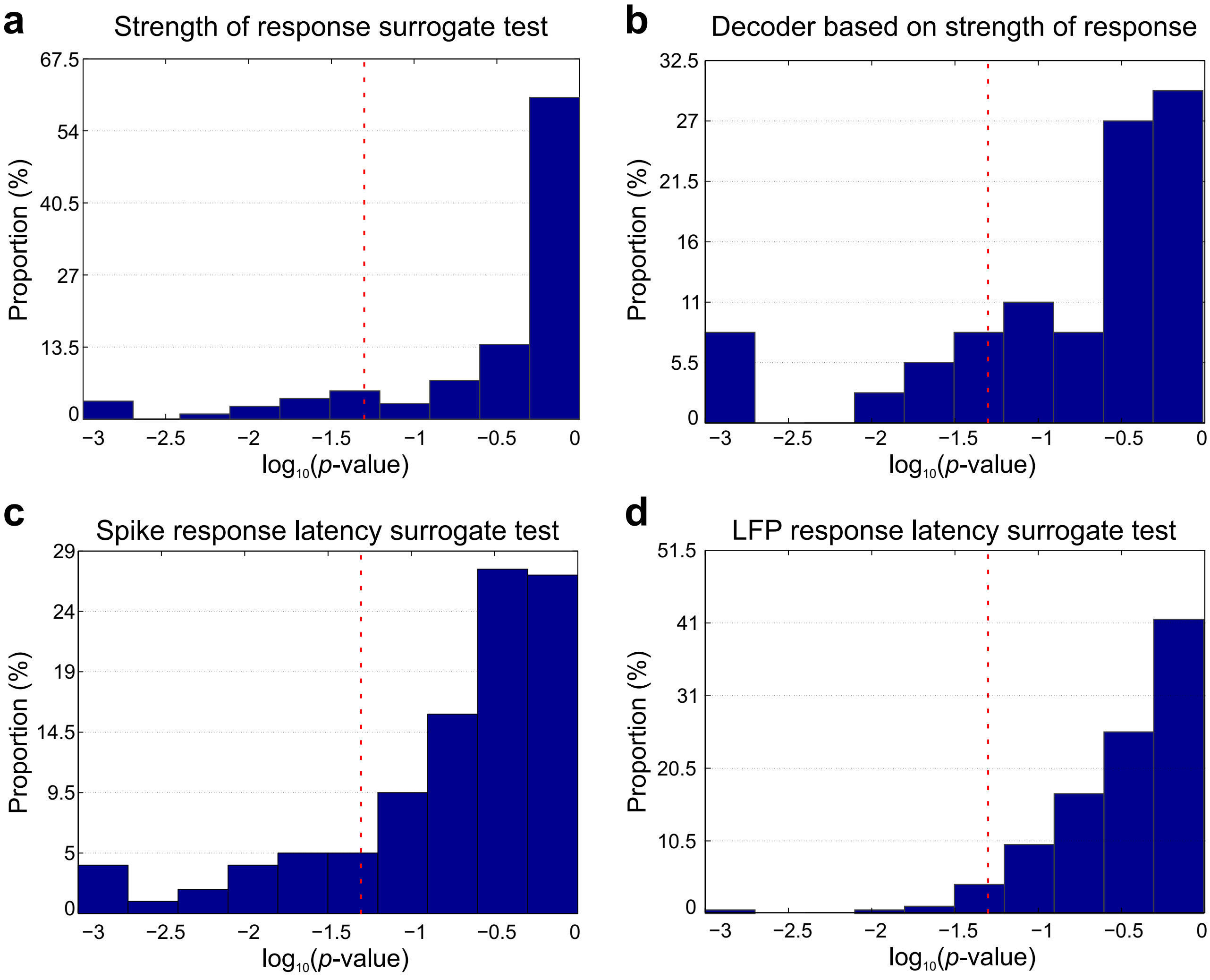

Supplementary Figure 1. Distribution of *p*-values. P-values for the surrogate tests using the strength and latency of the spike responses (top and bottom left, respectively) and latency of the LFP responses (bottom right). The top right plot shows the distribution of *p*-values for the decoding analysis. Vertical dashed lines represent the significance level of 0.05. The percentage of significant comparisons for tests in **a** to **d** was 14%, 22%, 19%, and 4%, respectively.

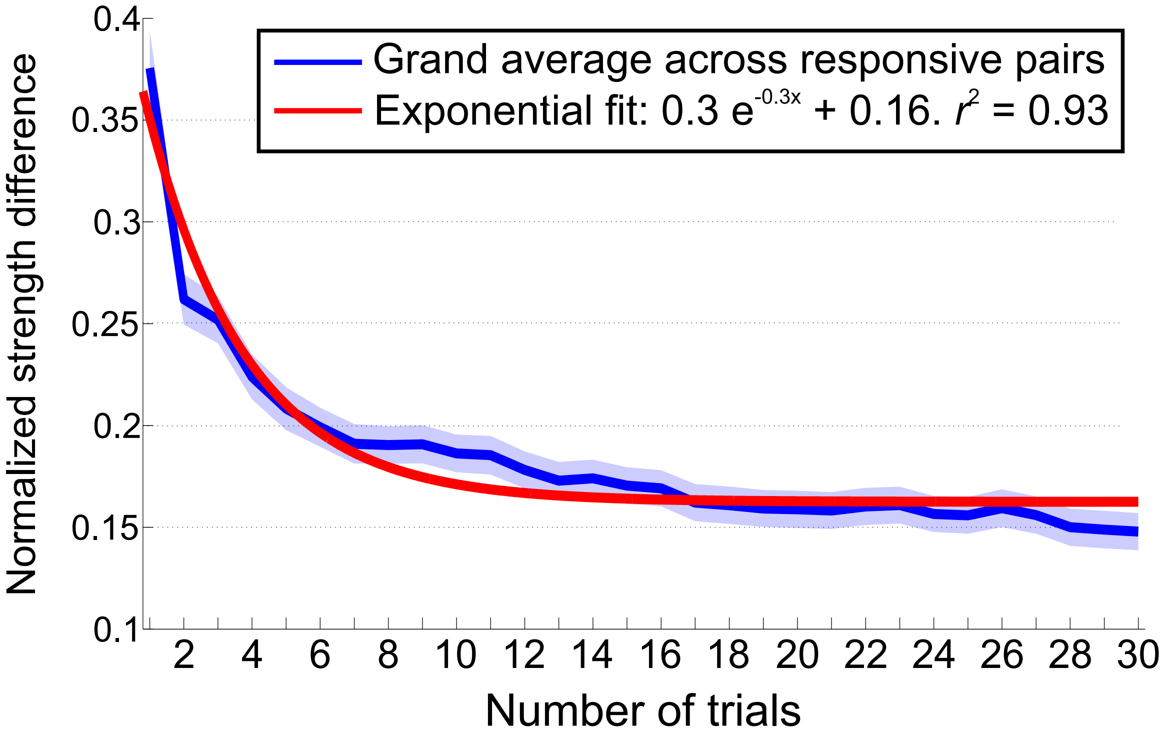

Supplementary Figure 2. Normalized strength difference estimation as a function of the number of trials used. Grand average of the normalized response strength difference across 208 responsive pairs. Data can be fitted with an exponential function, showing that the estimate became stable when using at least 18 trials (less than 1% change).

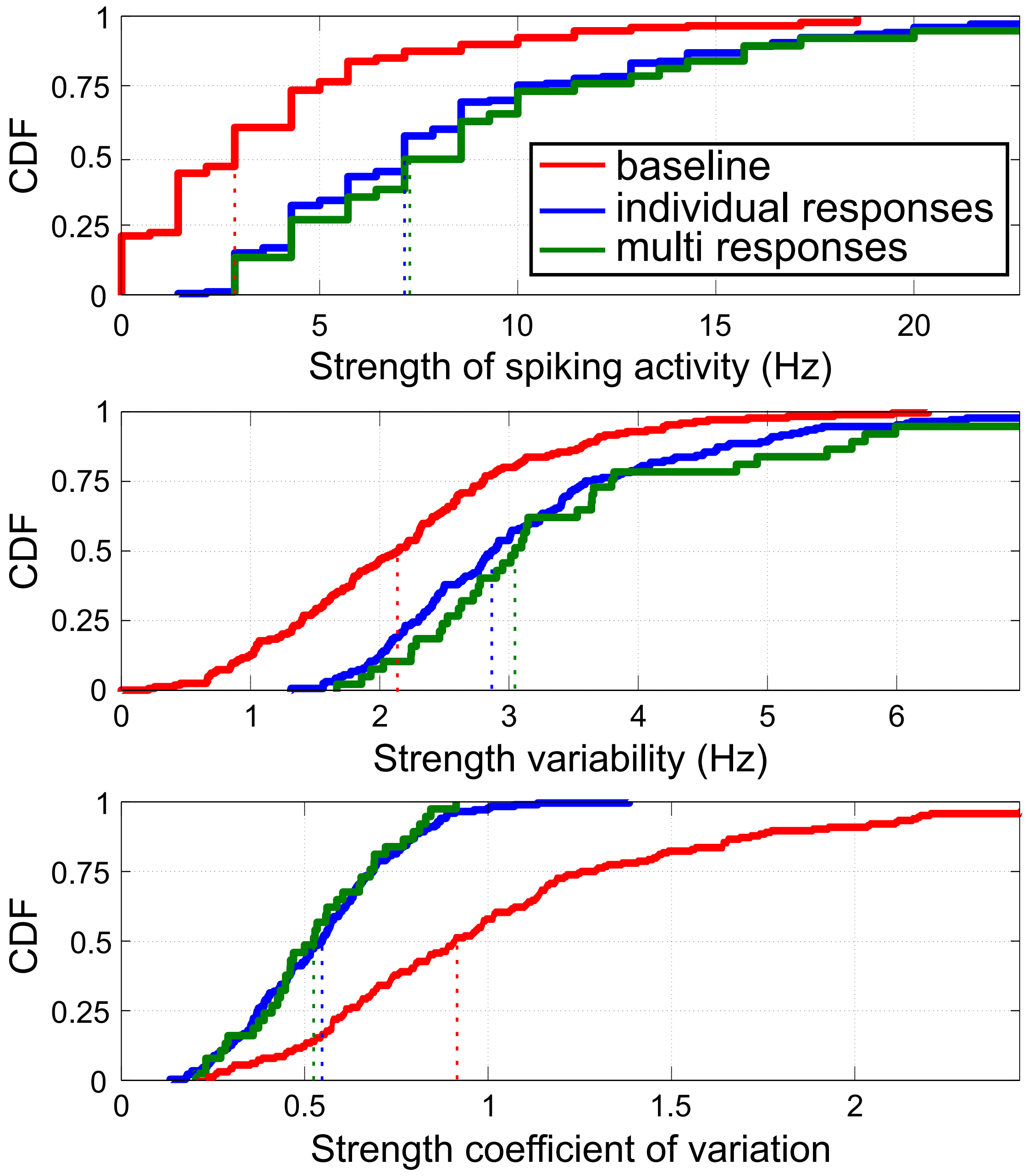

Supplementary Figure 3. Single- vs multi- responses strength comparison. From top to bottom, cumulative distribution functions (CDF) of mean, variability and coefficient of variation for the strength (spike count). In each case, strength was computed during baseline, and response period, which in turn could be for individual responses (n = 165) or for multiresponses (n = 37). The latter were computed by collapsing the trials of all the responses in a multi-responsive unit before computing each particular measure (see Methods). Vertical dotted lines denote the median of each distribution. There was a significant spiking activity difference between baseline and the individual responses (Kolmogorov Smirnov test, $p \sim 10^{-15}$), which was expected from the response criterion used. Moreover, there was no difference between the individual and the multiple response distributions (Kolmogorov Smirnov test, $p = 0.86$). Similarly, for the variability of the spike count across trials, there were differences between baseline and individual responses (Kolmogorov Smirnov test, $p \sim 10^{-9}$), but not between individual and multiple responses (Kolmogorov Smirnov test, $p = 0.37$). The same pattern was found for the coefficient of variation, showing a significant difference between baseline and individual responses (Kolmogorov Smirnov test, $p \sim 10^{-18}$), but not between individual and multiple responses (Kolmogorov Smirnov test, $p = 0.97$). Note that in this case, the baseline distribution had a median close to 1, which is consistent with a Poisson process. In contrast, the responses show a coefficient of variation smaller than 1, indicating that the spike count variability was smaller than the spike count average.

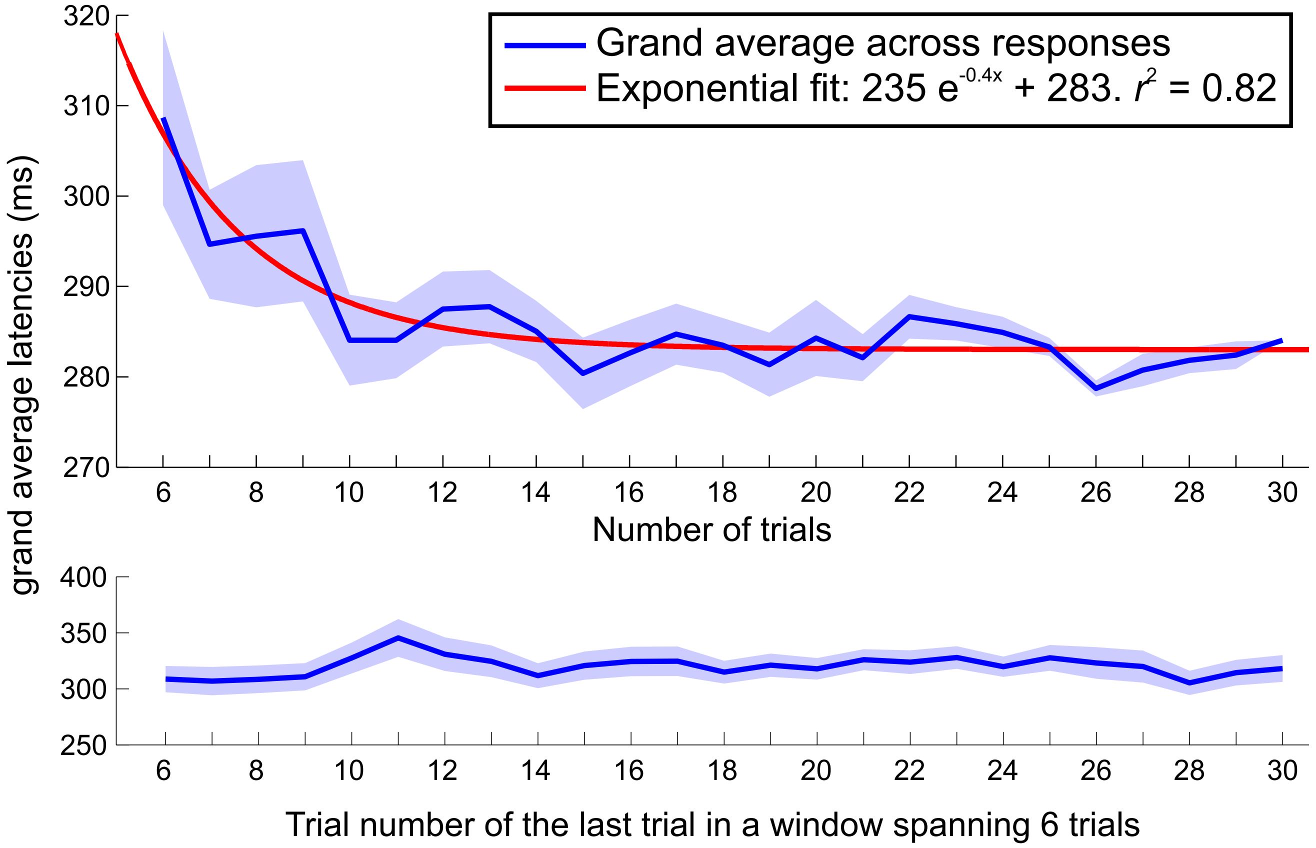

Supplementary Figure 4. Spike latency estimation as a function of the number of trials used. The top panel shows the grand average of the spike latencies across responses. Data can be fitted with an exponential function, showing that the estimate became stable when using at least 12 trials (less than 1% change). The bottom panel shows the estimation of the latencies using a sliding window of 6 trials (Methods), where no trend is observed. Comparing the estimation based on trials 1 to 6 with the one on trials 20 to 25 gave no significant differences (n = 138, two-sided paired sign test, p = 0.93). This confirms that the estimation bias seen using 6 trials is due to the window length and not to systematic changes (e.g. due to habituation) during the recording session.

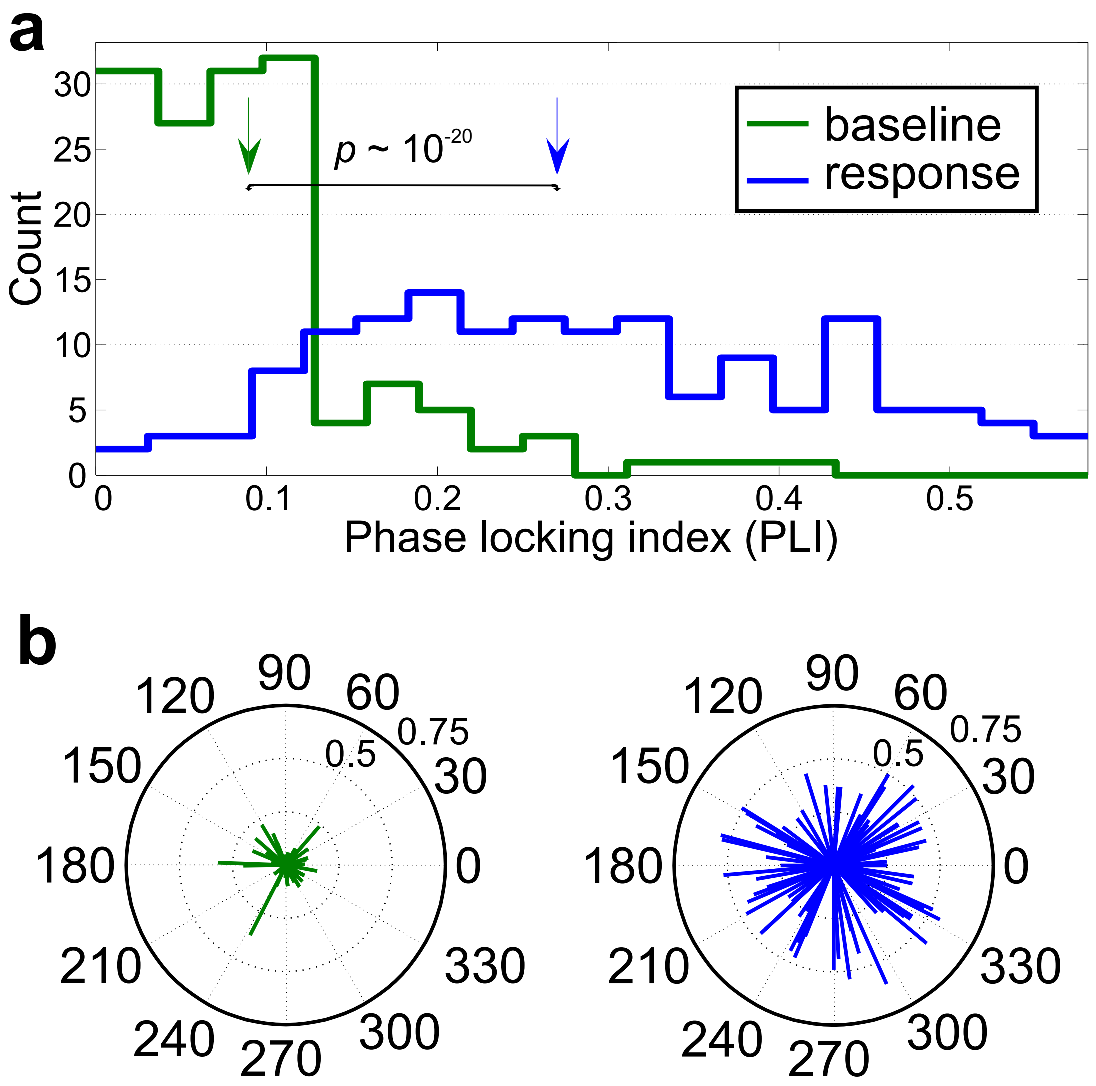

Supplementary Figure 5. Phase locking analysis. (**a**) Histograms of the phase-locking values computed for the individual spike responses. There was a significant difference between the distribution of phase-locking values in the response (n = 150) and baseline (n = 146) epochs (one-sided paired sign test, $p \sim 10^{-20}$). (**b**) Mean phase vectors (preferred phase) for the 82 significant responses in the response period (Rayleigh test, $p < 0.05$). Color coding is the same as in **a**. Only 10 out of 82 (12%) mean phase vectors were significant in the baseline period. Interestingly, the mean phase vectors in the response period span a wide range of preferred phases (Rayleigh test, $p = 0.15$).

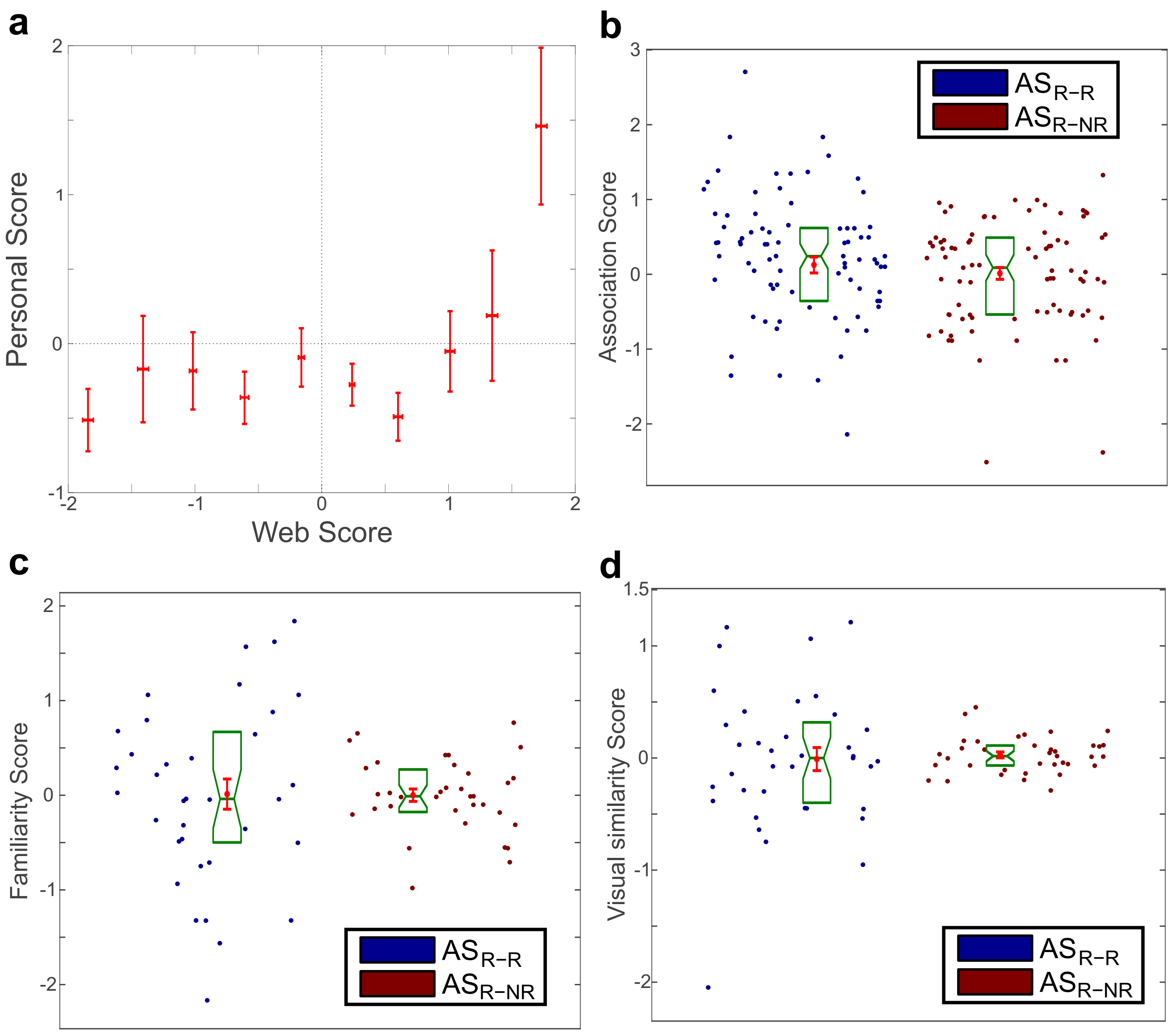

Supplementary Figure 6. Web-association metric and comparison with other metrics. (**a**) Comparison between the web association score and the personal associations scores provided by the subjects. There was a significant correlation between these metrics (Spearman correlation, n = 131, $\rho = 0.24$, $p = 5.9 \times 10^{-3}$). (**b**) Web-based mean association scores for pairs segregated into the same category pairs (see Methods for details on the definition of semantic categories). Response-eliciting pairs have a significantly larger association score (n = 85, one-sided paired sign test, $p = 0.04$). Same conventions as in **Figure 6a**. (**c**) Same as (**b**) but using a familiarity score, where no significant differences were found (n = 35, paired sign test, $p = 0.63$). (**d**) Same as (**c**) but using a visual similarity score, where no significant differences were found (n = 37, paired sign test, $p = 0.74$).

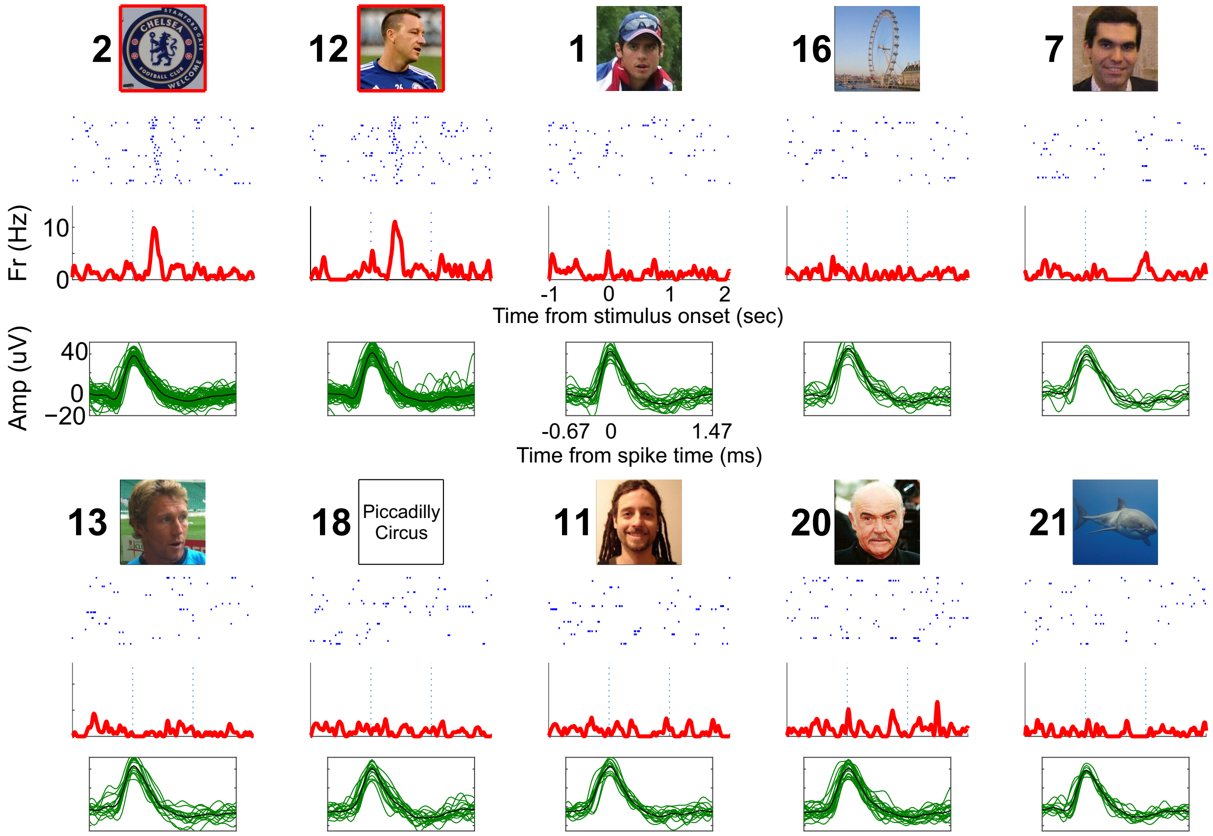

Supplementary Figure 7. Exemplary units with unitized responses. For each stimulus, the raster plot, instantaneous firing rate and spike shapes in the response period, are shown. Multi-response unit from the left hippocampus. Decoding performance was 48% (p = 0.53). The neuron responded to the Chelsea badge and to a picture of (Chelsea's captain) John Terry. Eight non-responsive stimuli shown in the session are also presented. The association scores for this unit were $AS_{R\text{-}R}$= 1.8 and $AS_{R\text{-}NR}$= 0.2, showing that the items the neuron fired to are highly associated. Due to copyright issues, the images presented here are similar to the ones actually presented to the subjects. Copyright notes: Picture 11 is a self-portrait from Hernan G. Rey (first author of the paper). Picture 7 is a self-portrait from Antonio Valentin (co-author of the paper). Picture 2 was cropped from “Chelsea Football Club” by Russell Trebor, licensed under CC BY-SA 2.0. Picture 21 was cropped from “Great White Shark” by Elias Levy, licensed under CC BY 2.0. Picture 13 was cropped from “Jonny Wilkinson 2009 08 12 3 Whitton twickenham england training” by elyob, licensed under CC BY-SA 2.0. Picture 16 was cropped from “London Eye from Westminster Bridge” by Kalaha, licensed under CC BY-SA 1.0. Picture 20 was cropped from “Sean Connery 1999” by Georges Biard, licensed under CC BY-SA 3.0. Picture 1 was cropped from “Alastair cook bowl” by Blnguyen, licensed under CC BY-SA 3.0. Picture 12 was cropped from “John Terry 01 Chelsea vs AS-Roma 10AUG2013” by Warrenfish, licensed under CC BY-SA 3.0.

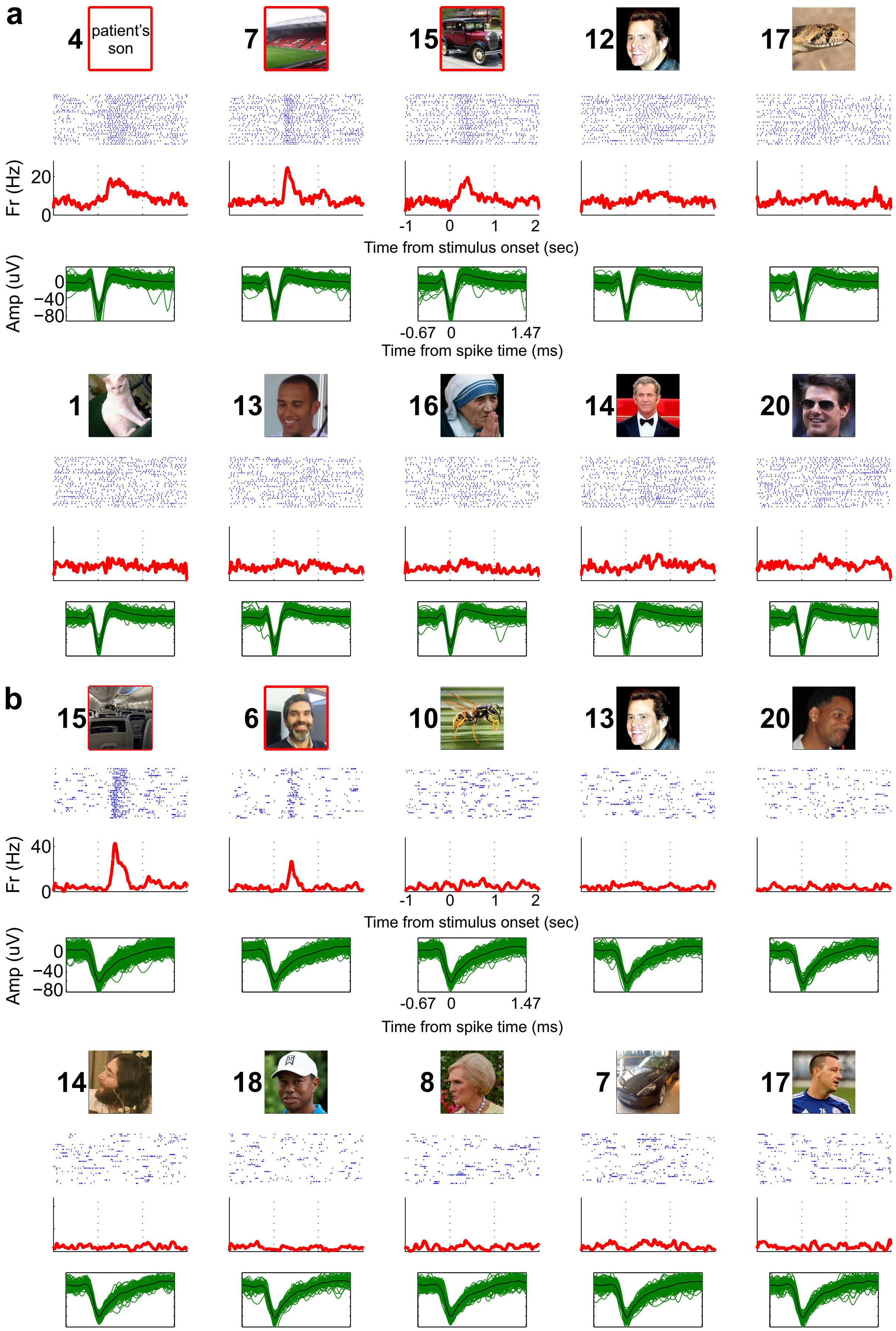

Supplementary Figure 8. Two exemplary units with significant decoding performance (i.e. showing differences in the responses). Continue on next page.

Supplementary Figure 8. (Continue from previous page). Same conventions as in **Supplementary Figure 7**. (**a**) Multi-response unit from the right hippocampus. Decoding performance was 47.8% (p = 0.025). Stimulus 4 was a picture of the patient's son, whereas the other two responses were for the Liverpool stadium and an old car. Seven non-responsive stimuli shown in the session are also presented. The association scores for this unit were AS_{R-R}= -2.5 and AS_{R-NR}= -0.5, showing that the items the neuron fired to are not associated. Due to copyright issues, the images presented here are similar to the ones actually presented to the subjects. Copyright notes: Picture 15 was cropped from “1928 Model A Ford” by Richard Smith, licensed under CC BY 2.0. Picture 12 was cropped from “Jim Carrey Cannes 2009” by Georges Biard, licensed under CC BY-SA 3.0. Picture 17 was cropped from “Gopher Snake Head Lateral wray” by Oregon Department of Fish & Wildlife, licensed under CC BY-SA 2.0. Picture 14 was cropped from “Mel Gibson Jodie Foster Cannes 2011” by Georges Biard, licensed under CC BY-SA 3.0. Picture 16 was cropped from “Mother Teresa holy” by Suma Iyer, licensed under CC BY-SA 4.0. Picture 1 was cropped from “White cat sitting” by Jack11 Poland, licensed under CC BY-SA 3.0. Picture 7 was cropped from “The Kop, Anfield” by Robert Cutts, licensed under CC BY 2.0. Picture 13 was cropped from “Lewis Hamilton portrait” by Ben Novakovic, licensed under CC BY-SA 2.0. Picture 20 was cropped from “Tom Cruise (34450932580)” by Eva Rinaldi, licensed under CC BY-SA 2.0. (**b**) Multi-response unit from the right hippocampus. Decoding performance was 88.3% ($p < 10^{-3}$). Stimulus 15 was the interior of an airplane cabin and the other responsive stimulus is a picture of Dr Antonio Valentin, one of the clinicians interacting with the patient. The association scores for this unit were AS_{R-R}= -2.9 and AS_{R-NR}= -0.69, showing again that these responsive concepts are not associated with each other. Copyright notes: Picture 7, “Aston Martin Virage” by Benoit cars, licensed under CC BY-SA 2.0. Picture 6 is a self-portrait from Antonio Valentin (co-author of the paper). Picture 13 was cropped from “Jim Carrey Cannes 2009” by Georges Biard, licensed under CC BY-SA 3.0. Picture 14 was cropped from “John rehearses Give Peace A Chance” by Roy Kerwood, licensed under CC BY 2.5. Picture 8 was cropped from “Mary Berry at Chelsea Flower Show - 2017” by Stephen Reed, licensed under CC BY-SA 2.0. Picture 10 was cropped from “Vespula germanica Horizontalview” by Richard Bartz, licensed under CC BY-SA 2.5. Picture 20 was cropped from “Will Smith 2” by Taís Melillo, licensed under CC BY-SA 2.0. Picture 15 was cropped from “Airplane Cabin 1 2017-06-18” by FASTILY, licensed under CC BY-SA 4.0. Picture 17 was cropped from “John Terry 01 Chelsea vs AS-Roma 10AUG2013” by Warrenfish, licensed under CC BY-SA 3.0. Picture 18 was cropped from “Tiger Woods 2018” by Keith Allison, licensed under CC BY-SA 2.0.

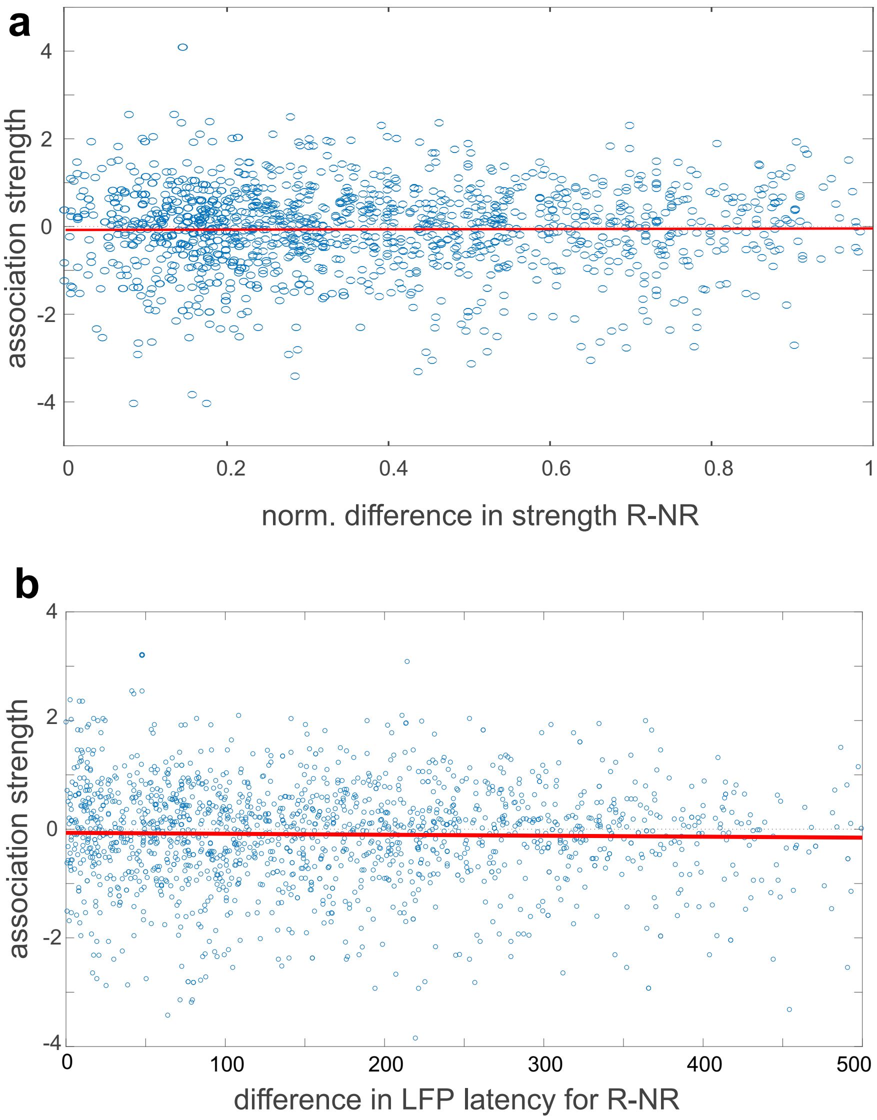

Supplementary Figure 9. Association score correlation analyses for pairs of responsive and non-responsive stimuli. (**a**) Association score as a function of the normalized difference in strength for R-NR pairs. There was no correlation between them (n = 1270, Pearson correlation, $r = 0.01$, $p = 0.74$), and it was significantly different to the one for the R-R pairs (Fisher Z transformation, $p = 9.2 \times 10^{-3}$). (**b**) Same as (**a**) but for the difference in LFP latency. No correlation was observed (n = 1157, Pearson correlation, $r = -0.05$, $p = 0.08$), while being significantly different to the one for the R-R pairs (Fisher Z transformation, $p = 1.5 \times 10^{-2}$).

Session number	1	2	3	4	5	6	7	8	9	10	11	12	13	14	15	16	17	18	19	20	21	Total
# neurons in Hippocampus	12	9	3	1	1	2	1	1	1	1	1	5	2	1	1	6	5	5	5	4	3	70
# neurons in Amygdala	0	0	0	0	0	0	0	0	0	0	3	0	0	0	0	3	2	2	1	0	0	11

Supplementary Table 1. Number of responsive units on each area (amygdala and hippocampus) per experimental session.
